# The UK’s expanding global reach for seafood over 120-years

**DOI:** 10.1007/s11160-025-09942-x

**Published:** 2025-04-18

**Authors:** Zoe F. J. Heard, Callum M. Roberts, Ruth H. Thurstan

**Affiliations:** Centre for Ecology and Conservation, University of Exeter, Cornwall, TR10 9FE UK

**Keywords:** Seafood consumption, Globalisation, International trade, Consumer footprint, Global fisheries, Historical ecology

## Abstract

Seafood markets have become increasingly internationalised over the course of the twentieth century, induced by expanding footprints of fishing fleets, improved communication and transport infrastructure, and trade agreements. We compiled archival UK seafood import data from UK Government, SEAFISH and FAO sources to track the expansion of the UK’s global reach for seafood products from 1900 to 2020. UK domestic fisheries landings declined from the 1970s following overexploitation and regulatory reforms, leading to a growing dependence on fish catches outside national waters and the international seafood trade-network. The volume of reported seafood imports increased by 6.4-fold from 1900 to 2020, overtaking domestic landings in 1985, with the species composition of these imports reflecting the palette of UK consumers, i.e., for the ‘big 5’ of cod, haddock, salmon, tuna and prawns, alongside agri/aquaculture industry demands for fishmeals/oils. The number of reported countries from which the UK imported seafood increased from five in 1900 to eighty-nine in 2020, covering all continents. The average distance seafood was imported increased by between 18 and 32%, from 2980 km (1900) to ~ 3520–3940 km (2020) (UK Government and SEAFISH data respectively), demonstrating the increasing geographic spread of UK demand. These results accentuate the need for stringent domestic fisheries management to recover local fish stocks, consumer diversification beyond the ‘big 5’, and for improved collaborative international fisheries governance to mitigate the potential for serial depletion of popular food fish.

**Graphical abstract:**

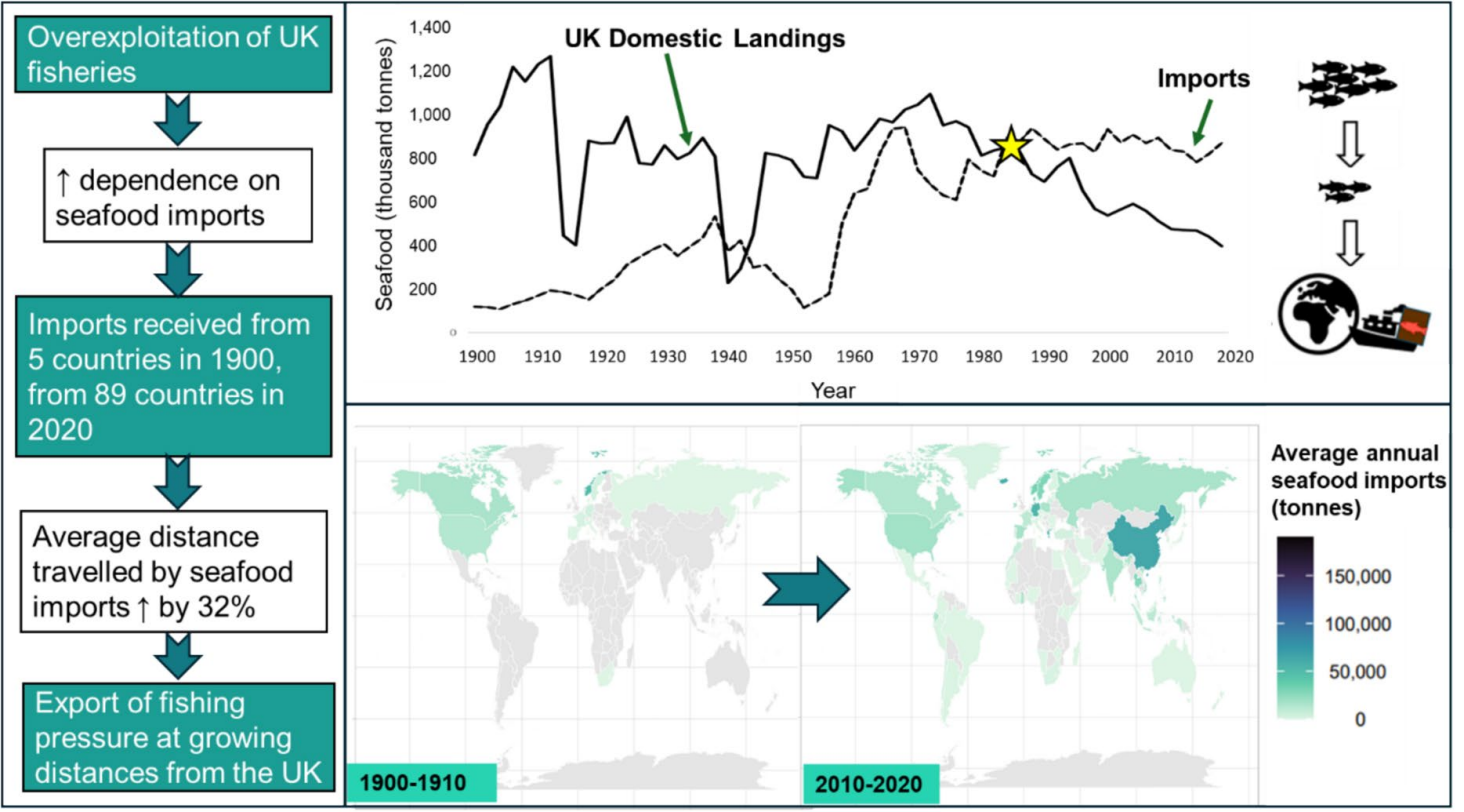

**Supplementary Information:**

The online version contains supplementary material available at 10.1007/s11160-025-09942-x.

## Introduction

Seafood products are important for the food security and micronutrient needs of billions of consumers across the globe (FAO 2022; Gephart and Pace 2015; Peterson and Fronc 2007). Globally, we are eating more aquatic foods than ever before with current statistics suggesting per capita consumption is at 20.2 kg/year, over double the consumption 50 years ago (FAO 2022). Consumption growth, facilitated and encouraged by fast growing populations, rising incomes, seafood commoditisation, and the promotion of marine products for their health properties (Belton et al. 2020; Rood and Schechter 2007; Thurstan and Roberts 2014), in conjunction with mismanagement of fisheries, has led to widespread declines and collapses of marine fish stocks (Mullon et al. 2005; Thurstan and Roberts 2014; Watson and Pauly 2001). In many countries, local capture fisheries are no longer able to satiate domestic markets (D’Odorico et al. 2014; Godfray et al. 2010; Taylor et al. 2007).

### Trends and pressures on UK domestic fisheries

The UK’s capture fishing industries were among the most productive in the world in the early to mid-twentieth century (Tunstall 1968). However, increased extraction of fishery resources driven by technological innovations and laissez-faire management led to a national fisheries crisis in the latter part of the century (Dickey-Collas et al. 2010; Holm 2012; Holm et al. 1996; Karlsdottir 2005; Kerby et al. 2012; Reid 2010). Declines in both demersal and pelagic North Sea and North Atlantic fisheries prompted reforms in European fisheries management, including the introduction of rebuilding targets and catch restrictions under the Common Fisheries Policy from the 1980s and 90s (Harrison et al. 2023; Kerby et al. 2012; Roberts 2007; Thurstan et al. 2010; Whitmarsh et al. 1995). Furthermore, the establishment of 200-mile Exclusive Economic Zones (EEZs) from the mid-1970s to the early 1980s meant that UK fishing fleets were progressively excluded from productive distant-water fishing grounds, e.g., around Iceland (Harrison et al. 2023; Mulazzani & Malorgio 2015). In recent years, climate change driven range shifts of target species, namely cod (Engelhard et al. 2014) and mackerel (Jolly 2013), in addition to continued fishing pressure (Engelhard et al. 2014), has posed a further threat to the persistence of and access to traditional UK fish stocks (Godfray et al. 2010). These drivers of decline in UK fisheries ultimately resulted in reduced seafood production and self-sufficiency (Mulazzani & Malorgio 2015).

### Exporting overfishing

Globalisation has been pivotal in improving food security by making available a wider variety of foodstuffs and buffering against local supply problems (Crona et al. 2015; Gephart and Pace 2015; Graziano et al. 2018). From the 1950s, increasing seafood demand and depletion of local fish stocks promoted the growth of heavily subsidised distant-water fishing fleets, particularly in Europe and Northeast Asia, which increased their exploitative effort in the Global South and on the high seas (Belhabib et al. 2015). The establishment of Euro-African fishing agreements from the 1970s meant Europe’s distant-water fleets could pay fees to African coastal states for access to fishing resources, mainly on unfavourable terms to the host nations (Belhabib et al. 2015; Iheduru 1995; Swartz et al. 2010). Long distance trade also buffered against local fish supply deficits. This activity is hardly new, with archaeological evidence suggesting the trade of Viking-era (800–1066 CE) Arctic cod from Northern Norway to mainland Europe, over 1000 kms away (Star et al. 2017). However, international trade has grown rapidly in volume and reach over the past century and the magnitude of today’s global interconnectedness and interdependence is unprecedented; seafood products are now among the most highly traded commodities across the world (Crona et al. 2016; FAO 2022; Gephart & Pace 2015).

Trade intensification has been facilitated by rapid advances in technology and transport, processing and communication infrastructure over the course of the twentieth century (Asche et al. 2015; Belton et al. 2020; Rood and Schechter 2007). Expansion of international collaboration and trade agreements, neoliberal economic policies, and the creation of intergovernmental organisations such as the World Trade Organisation, the United Nations and the European Union in the mid to late twentieth century further acted to increase flows of commodities by removing barriers to trade (Alder and Watson 2007; FAO 2022; Harrison et al. 2023; Rood and Schechter 2007). The latter part of the century hence saw the increased and accelerated access to distant markets and a newfound dependence on a diverse supply of marine resources from locations across the world (FAO 2022; Taylor et al. 2007).

Today, an estimated 81% of seafood by volume eaten in the UK is imported from overseas (Wolf et al. 2020), and self-sufficiency for commercially important seafood groups such as whitefish, salmonids, crustaceans and pelagics is below 30% (WWF 2022). British seafood preferences are generally restricted to a narrow range of species, often referred to as the ‘big 5’—cod, haddock, salmon, tuna and prawns—making up ~ 80% of all seafood consumed (Harrison et al. 2023; Peterson & Fronc 2007; Tetley 2016). The restricted seafood palette of UK consumers is thought to be a product of historically conservative patterns of fish consumption, reinforced by the way fish is sold and filleted/tinned and ready to cook (Tetley 2016). Socio-demographic attributes, including age, wealth, and educational level, are also known to influence fish consumption preferences (Future Foundation 2014; Jennings et al. 2016; Thurstan 2022; Tunstall 1968). Large quantities of fishmeals and oils are also imported into the UK for use in agriculture and aquaculture (Harrison et al. 2023; Tunstall 1968). Henceforth, in this study, this group of species and products will be referred to as the ‘big 6’.

There is a growing eagerness, among researchers in the realm of sustainable development, to assess the impact of globalisation on consumer footprints, i.e., the environmental impact of consumer driven appropriation of the world’s natural resources (Sala and Castellani 2019). For example, D’Odorico et al. (2014) and Gephart and Pace (2015) investigated the role of the global food trade network in feeding humanity from the late twentieth century and found that the globalisation of resources led to the externalisation of environmental impacts, i.e., exerting pressure on resource systems across more areas of the world. The decline of fish catches and stock collapses, e.g., of Peruvian anchoveta (*Engraulis ringens*) in the 1970s (Thorpe and Bennett 2001), of South African and Namibian pelagic fisheries in the 1980s (Crosoer et al. 2006) and of Chilean hake (*Meluccius gayi*) in the 1990s (Schurman 1996), have also been attributed in-part to globalisation as countries expand their fishing fleets and grounds and increase use of destructive fishing practices to meet global consumer demand (Alder and Watson 2007).

In this study we draw on archival UK fish import data covering the past 120 years to understand how the UK’s global reach for marine seafood products expanded since 1900. This is especially pertinent in the face of Brexit and the introduction of barriers to trade (e.g., tariff restrictions) which pose a serious threat to the UK’s seafood supply chain from Europe (Symes and Phillipson 2019). It is also pertinent at a time when the UK is striving for net-zero as carbon emissions associated with industrial fishing fleets and seafood cargo transportation are an environmental concern related to expanding trade networks (Halevy and Trewern 2023; Sonesson et al. 2010; Tyedmers et al. 2005). We also aim to shed light on the growing UK dependency on seafood imports and its socio-ecological repercussions on marine ecosystems, food security and human rights, in producing nations.

The EU was the leading importer of seafood in 2020 (namely Spain, Denmark, France, Germany and the Netherlands), followed by China, the USA, Japan, Thailand and South Korea (FAO 2020), hence dependency on the global seafood trade network is by no means confined to the UK. However, the UK was listed as the 13th largest importer in 2020 (FAO 2020), and in the top ten countries most central to the seafood trade network in 1994 (ranking no.1) and 2012 (ranking no.8) (Gephart and Pace 2015), therefore gaining a comprehensive understanding of the evolution of the UK’s global reach is of high relevance for global sustainable marine resource management.

### Sources & methods

UK domestic fisheries landings and seafood import data were retrieved from the Marine Management Organisation’s ‘UK Sea Fisheries Statistics Archive’ (1890–2011) and ‘UK Sea Fisheries Annual Statistics’ (2012–2020) online repositories- henceforth, these data sources are referred to as the archive data (MMO 2014, 2021). The FAO FishStatJ database of fishery statistics (FAO 2020) contained total UK import data from 1976 and was used to corroborate the accuracy of the archival seafood import data from this date onwards; the non-parametric Wilcoxon rank-sum test (Wilcoxon 1945) was used to test the null hypothesis (H_0_) that the two datasets have equal medians (R Core Team 2023). Recent data on seafood imports by country of export, from 2011 to 2019, were retrieved from the SEAFISH Trade and Tariff Tool (SEAFISH 2023), originating from His Majesty’s Revenue and Customs. These data did not span the entire time-series but offered an interesting comparison to the archive data as its higher resolution illuminates past ambiguities of historical records. Due to the extensive time-period and quantity of data available, we extracted data biennially, i.e., for every second year from 1890 to 2020, though data from the 1890s were later excluded due to poor resolution. Data from annual reports are provisional until the following year, hence the data were taken from the consecutive years’ report. Up to 1975, landings were recorded in UK hundredweights (cwts) rather than metric tonnes; therefore the final dataset was standardised using the following conversion factor: 1cwt = 0.0508 tonne.

### Domestic landings versus imports

The total weight of marine finfish and shellfish landed into UK ports, including from foreign vessels, and total seafood imports coming into the UK were digitised to allow comparison of the UK’s dependence on imports versus home supplies. UK shellfish landings data were not available in weight between 1900 and 1961 in the archive reports so ICES data on UK shellfish landings were used to supplement the dataset over these years (ICES 2023). It should be noted that tonnages of domestic landings correspond to the weight of head on, gutted fish (Thurstan and Roberts 2014) whereas imported seafood weights were recorded as processed. Exact comparisons are therefore not possible (Bjørndal et al. 2015). Seafood import quantity data were preliminarily tested for autocorrelation using the Durbin Watson (DW) test (Durbin and Watson 1950) (‘tseries’ package) (R Core Team 2023). The DW statistic (DW = 0.28) indicated strong autocorrelation (values near 2 are considered optimal), hence the modified ‘Hamed and Rao (1998) Variance Correction Approach’ Mann–Kendall (MK) test (‘modifiedmk’ package) which is robust in the presence of serially correlated data was used to detect the presence of a statistically significant trend across the time-series (R Core Team 2023). The MK test tests the H_0_ that there is no significant trend in the series, for a given significance level (α = 0.05) i.e., if the *p* value < 0.05, the H_0_ should be rejected, the Kendall’s tau (*τ*) statistic determines the strength and direction of the trend; it is one of the most popularly applied tests in a variety of environmental fields to detect significant trends in time-series (Meggiorin et al. 2021; Yue and Wang 2004).

### Measuring the expansion of the UK’s global reach for seafood: exporting countries & weighted average distances

Data on seafood imports into the UK were digitised and tabulated by country of export, i.e., for each country that exported seafood to the UK in a given year, export weights for different seafood products were summed. Decadal averages were then calculated for each exporting country and world heatmaps of seafood sources were created in R (R Core Team 2023) using ‘ggplot2’ and ‘dplyr’ packages. Where sums of seafood imports from different countries are discussed in the results, these are estimated by doubling seafood import quantities from biennially extracted data. The total number of countries that the UK imported from were also totalled each year and plotted using the ‘ggplot2’ package (R Core Team 2023).

To calculate the distance between London and capital cities across the globe, the site: ‘https://www.distance.to/’ was used. This site converts place names into coordinates and the Haversine formula (an accurate methodology to compute distances between two points on a sphere using the latitude and longitude of these points (Kettle 2017)) is applied to establish distance (km). We then calculated weighted average distances using the total distance between exporting countries’ capital cities and London, in a given year, and total weight (tonnes) of seafood coming from these countries, with the ‘weighted.mean’ function (R Core Team 2023). Biennial weighted averages were plotted from 1900 using the ‘ggplot2’ package in R. The time-series for total number of exporting countries and weighted average distance travelled displayed strong positive autocorrelation (DW = 0.18, DW = 0.07, respectively), therefore the modified MK test was used to detect any significant trends in these datasets (R Core Team 2023).

### Seafood products

Seafood product imports were digitised by country of export in the archive data from 1903 (when consistent product-specific record taking began) and included finfish, shellfish and fish/marine mammal products (MMO 2014, 2021). The recording of seafood products lacked species specificity and the grouping of ‘species’ henceforth is used loosely. For example: ‘salmon’ includes a number of species such as the Atlantic Salmon (*Salmo salar*), chum Salmon (*Oncorhynchus keta*) and sockeye (*Oncorhynchus nerka*); ‘cod’ include Atlantic cod (*Gadus morhua*), Pacific cod (*Gadus macrocephalus*) and Greenland cod (*Gadus ogac*); ‘haddock’ include only *Melanogrammus aeglefinus*, ‘tuna’ include skipjack (*Katsuwonus pelamis*), bigeye (*Thunnus obesus*), yellowfin (*Thunnus albacares*), bluefin (*Thunnus thynnus*) and albacore (*Thunnus alalunga*), and prawns include wild-caught cold-water prawns (*Pandalus borealis*) and (typically farmed) warm-water prawns such as king prawns (*Panaeus monodon*). Where ‘species’-level data were not available, imports were grouped into ‘miscellaneous’ fish and shellfish categories. Fish/marine mammal products included fish and whale meal and oil, whale bone and smaller quantities of miscellaneous products (e.g., fish roes); in 1975 the UK stopped importing whale meat and 1989 was the last year that fishmeals contained marine mammal products (MMO 2014).

Product specific import data were sporadic in the archive data as reporting styles changed through time. For instance, categories of fish/marine mammal products were only consistently reported from 1921 to 1943 and from 1959 to 2020. From 1965 to 2005, country of export product-specific information was available only for herring, salmon and fish/marine mammal products, all other species were grouped into ‘fresh/frozen’, ‘boneless’, ‘semi-preserved’ and ‘prepared/preserved’ demersal/pelagic categories which included cod, haddock, hake, tuna, brisling (sprat), mackerel, fish roes, plaice, sole and halibut, and filleted products such as fish fillets, fish fingers, tinned fish and fish paste (MMO 2014). Although the FishStatJ database did not report product specific imports by country of export, the higher resolution product-specific data available here from 1976 were used to account for part of this data gap (1965–2005) in the archive dataset (FAO 2020). FishStatJ data were collated and summed to replicate the same categories of seafood products as in the archive data, i.e., all tuna products were grouped, added, and categorised as tuna. For continuity of data resolution, the FishStatJ data were used from 1977 to 2020. Where data were comparable between the sources (from 1977 for herring, salmon and fish/marine mammal product imports, and from 2007 for cod, haddock, tuna, shrimp/prawn, sardine, mackerel and plaice imports), some discrepancies were found, with the datasets differing by on average 12.4%. This information, including possible explanations for larger discrepancies can be found in Online Resource 1. Seafood product imports were plotted by category in R (R Core Team 2023).

### Discrepancies in data reporting

In each UK Sea Fisheries Statistics report, seafood imports were reported by country of export and by an **‘**other countries’ category. The percentage of total imports that came from ‘other countries’ varied from year to year and a decadal average by weight of total seafood imports was calculated for them (Table 1). Further information on which countries were included in this category and what the export weight threshold was for inclusion in this category were not given in the reports.

**Table 1:** Percentage of UK seafood imports coming from ‘other countries’

Decade	%
1890–1900	63.4
1900–1910	8.9
1910–1920	1.8
1920–1930	4.5
1930–1940	27.2
1940–1950	31.9
1950–1960	23.5
1960–1970	1.6
1970–1980	8.7
1980–1990	20.2
1990–2000	19
2000–2010	13.6
2010–2020	18.2
2010–2020 (SEAFISH data)	0

In the 1890s, imports from ‘other countries’ equated to over 60% of total imports (Table 1), the poor data resolution for this decade compared to subsequent decades meant that it was excluded from analysis. For 2010–2020, data retrieved from SEAFISH had no ‘other countries’ category as all imports were assigned to a country of origin. We were unable to attribute imports from ‘other countries’ to any analyses related to the spatial expansion of the UK’s reach for seafood. Our results thus represent the minimum number of countries that the UK imported seafood and provide an estimation of the total weighted distance that seafood was imported from.

In some years, imports from countries were recorded as grouped, for example Iceland and Greenland exports were combined from 1900 to 1940, Soviet Union exports were combined from 1939 to 1990, and Belgium and Luxembourg exports were combined from 1993 to 1999; imports were assigned to the first country mentioned (i.e., Iceland, Belgium, and Russia for the Soviet Union). ‘Deep sea fisheries’ and ‘Northern/Southern Whale fisheries’ imports were not distinguished by country, so these imports were attributed to the ‘other countries’ category.

## Results

### Domestic seafood production versus imports

**Fig. 1:**
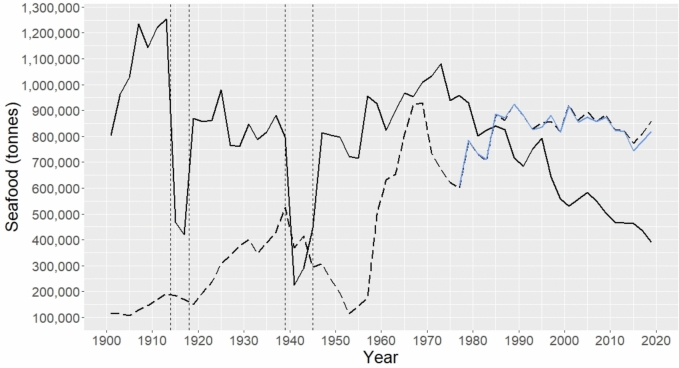
Trends in domestic landings of seafood (solid black line) and seafood imports into the UK (dashed black line: archive data; blue solid line: FishStatJ data) from 1900 to 2020. Note that imports represent processed weights while domestic landings represent pre-processed weights. Vertical dashed lines represent WW1 and WW2

UK domestic fisheries landings were consistently high through much of the twentieth century ranging from 700,000 tonnes to upwards of 1.2 million tonnes (mt) annually (Fig. 1). Exceptions to this were the two World Wars where landings crashed from a peak of 1.25 mt in 1913 to ~ 420–470,000 tonnes (WW1) and from 882,000 tonnes in 1937 to ~ 230–450,000 tonnes (WW2), though these crashes were short-lived and landings rebounded quickly (Fig. 1). From the early 1970s domestic landings declined by 64%, from 1.08 mt in 1973 to 391,000 tonnes in 2019, due to stock declines and collapses, and the introduction of regulatory measures and the creation of EEZs which excluded UK vessels from productive foreign waters (Harrison et al. 2023) (Fig. 1).

Annual seafood imports were relatively low (fluctuating between 100,000 and 550,000 tonnes) in the first half of the twentieth century but briefly surpassed domestic landings from 1941 to 1943 to supplement UK demand while UK fishing fleets were limited by diminished wartime fishing opportunities (Fig. 1). The dip in imports to less than 350,000 tonnes between 1945 and 1957 (Fig. 1) represents a change in reporting style from the data source, as imports of fish and marine mammal products (fish meals/oils and whale meat/oils) which contributed a large portion of UK imports in the other years, were not included in the reports. However, imports increased rapidly from 1957 and exceeded domestic seafood supply from 1985. Since then, the relative difference between domestic supply and imports has expanded, from 5% in 1985 to 75% in 2019. A strong statistically significant increasing trend over the seafood import time-series was found (modified MK, *τ* = 0.65*,* Z_c_ = 4.92, *p* < 0.05); imports rose by 6.4-fold (~ 636%) from 117,000 tonnes in 1901 to 860,000 tonnes in 2019 (Fig. 1). The archival and FishStatJ import data were well matched from 1977 (Fig. 1), with results from the Wilcoxon rank-sum test showing that they did not significantly differ (W = 256, *p* = 0.75), i.e., the H_0_ that the two datasets are equal could not be rejected.

### UK’s global reach for seafood

**Fig. 2:**
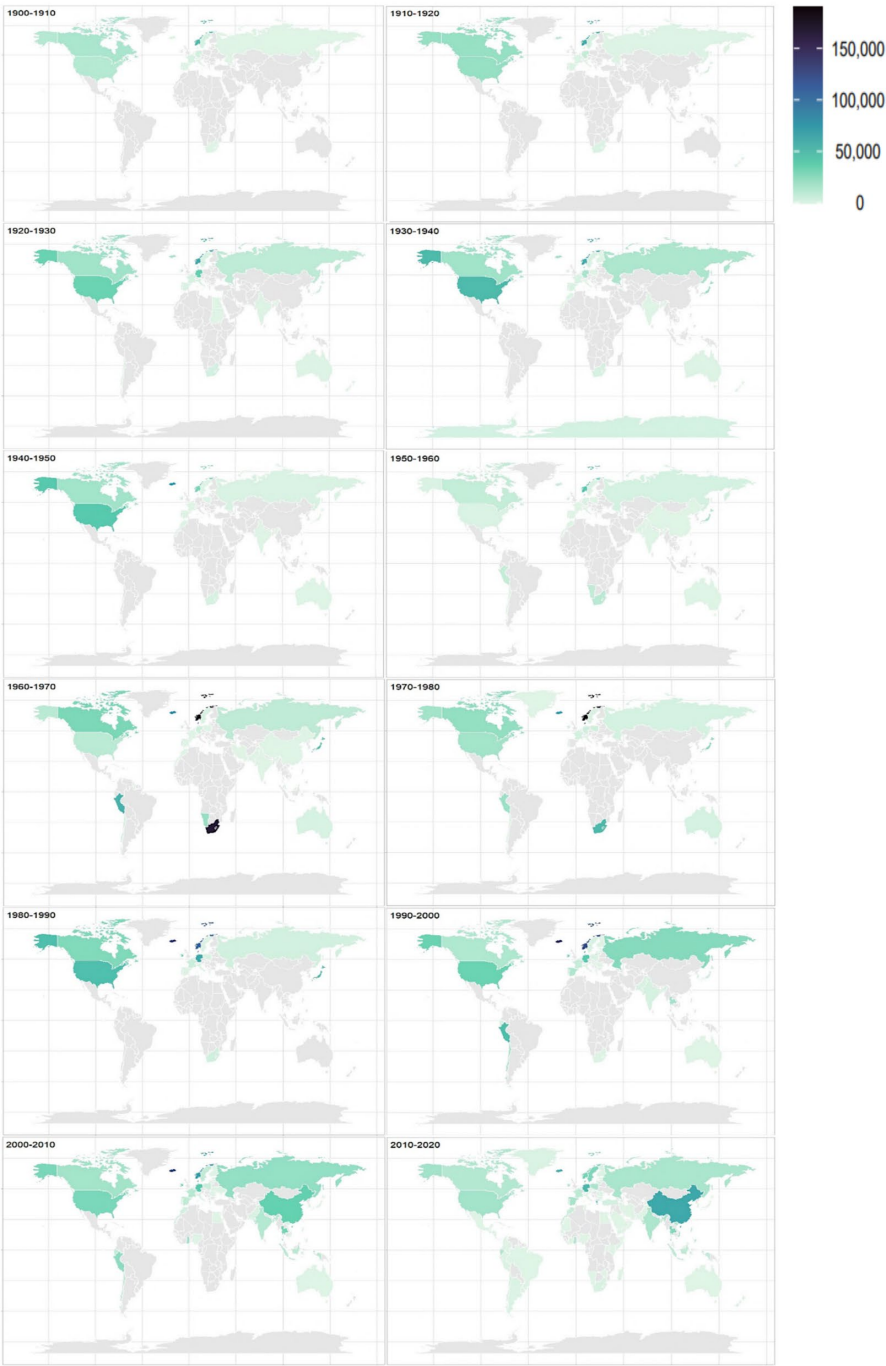
Origin of UK seafood imports covering 1900–2020. Colour gradient represents total annual tonnes of seafood exported to the UK (decadal average). SEAFISH data were used for 2010–2020

A total of 118 different countries exported an estimated 64mt of seafood products to the UK from 1900 to 2020. In the first half of the twentieth century imports originated predominantly from the global North, namely Europe, Russia, the USA and Canada (~ 10.2 mt, ~ 94% of imports by weight pre-1950), with Europe alone meeting 67% of demand (~ 7.2 mt pre-1950) (Fig. 2). The UK also imported from further afield, from South Africa, Egypt, Sudan, India, the Falkland Islands and the West Indies which were, at the time, part of the British Empire, as well as New Zealand, Australia (former colonies) and Japan (which formed good relations with the UK under the Anglo-Japanese Alliance in 1902) (Fig. 2). In 1933, the UK also imported close to 18,000 tonnes of whale oil from the Antarctic region (Fig. 2). From the 1950s, the UK increasingly became reliant on imports from Europe and more distant countries, with producing countries covering all populated continents (Fig. 2). From 1950 to 2000, 69% of imports by weight came from Europe (~ 20 mt over this fifty-year period), 9% from Africa (~ 2.6 mt), 8% from Asia (inc. Russia) (~ 2.4 mt), 8% from North America (inc. Canada, Greenland, Central America) (~ 2.2 mt), 6% from South America (~ 1.7 mt) and less than 1% from Oceania (~ 64,000 tonnes). In the past two decades, 2000–2020, Europe contributed to 58% of total UK seafood imports (~ 8.4mt) and Asian exports increased, contributing to 22% of imports over the twenty-year period (~ 3.2 mt), with China alone exporting an estimated 1mt (~ 6% of total imports) (Fig. 2). There were fewer exports originating from the North America (6%) and South America (5%), and roughly the same proportion of exports from Africa (9%) and Oceania (0.4%) compared to the latter half of the twentieth century.

The top 25 exporters (Table 2) contributed over 80% of total UK imports across the time-series. Europe, the USA, Canada, South Africa, India, Chile and Japan have been key to meeting the UK’s demand since the early twentieth century. Other producing countries became dominant seafood sources in later years, for example, China and Peru from 1950, Thailand and Mauritius from 1990, and the Seychelles and Ghana from 2000 (Table 2).

**Table 2:** Top 25 Countries exporting seafood to the UK (‘Seafood Exported’ figures are estimated by doubling exports from biennially extracted data), percentage contribution to total UK imports over the period 1900 to 2020, and the decade the UK started importing from each country

Country	Seafood exported (mt)	% of total imports	Decade
Norway	10.26	16.03	1900
Iceland	7.50	11.72	1900
Denmark	5.70	8.91	1900
USA	3.42	5.34	1900
Germany	2.79	4.36	1900
Holland	2.57	4.02	1900
Canada	2.54	3.96	1900
South Africa	2.45	3.84	1900
Faroe Isles	1.96	3.06	1940
Japan	1.88	2.94	1930
Peru	1.62	2.54	1950
Ireland	1.62	2.53	1920
Russia	1.19	1.86	1900
China	1.00	1.57	1950
Thailand	0.79	1.24	1990
Portugal	0.77	1.2	1900
Sweden	0.59	0.93	1900
France	0.56	0.87	1900
Spain	0.49	0.77	1900
Mauritius	0.48	0.76	1990
Belgium	0.44	0.69	1900
Seychelles	0.40	0.63	2000
Chile	0.36	0.56	1920
Ghana	0.35	0.55	2000
India	0.34	0.53	1920
Total	52.07	81.41	

**Fig. 3:**
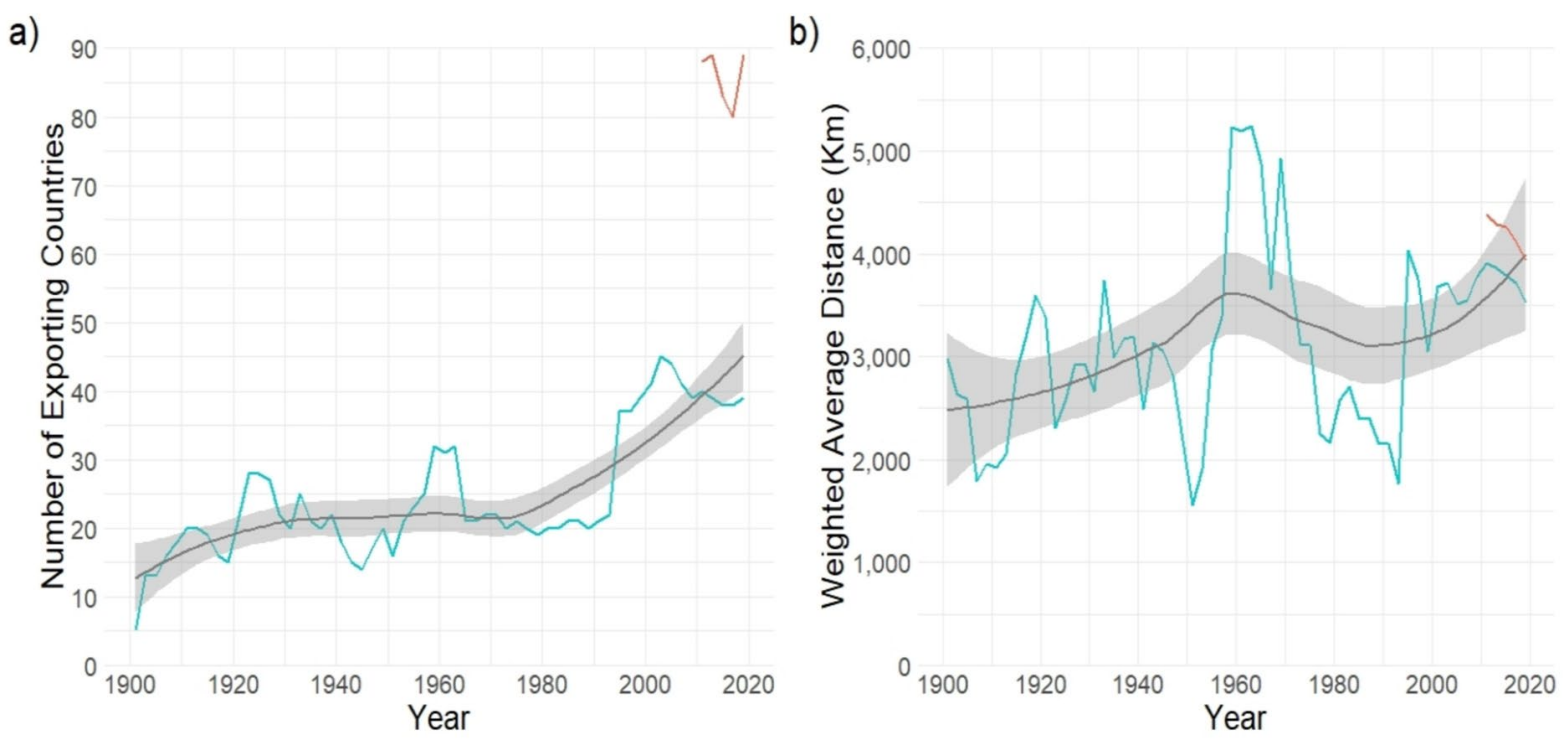
**a** the total number of countries exporting seafood to the UK, and **b** the weighted average distance seafood was exported from (1900–2020). Sources: Archived Sea Fisheries Statistical Tables (in blue), SEAFISH (in red). Black lines represent loess smooth regression lines and grey areas represent 95% confidence intervals, fitted using the “geom_smooth()” function in RStudio. Modified MK, *τ* = .48*,* Z_c_ = 3.33, p < 0.05 (**a**), and *τ* = .25*,* Z_c_ = 2.24, *p* < 0.05 (**b**)

The number of countries exporting seafood to the UK, based on the archive data, increased from 5 to 39 from 1900 to 2020 (Fig. 3a). A strong significant increasing trend (modified MK, *τ* = 0.48*,* Z_c_ = 3.33, *p* < 0.05) over the time-series was found, illustrating the increasing globalisation of seafood trade. The more detailed SEAFISH data covering 2010–2020 suggest that the actual recent number of exporting countries ranged between eighty and eighty-nine, over double those recorded in the archive reports (Fig. 3a). This is likely due to the greater rigour of data collection on international trade of goods by HM Revenue and Customs. When SEAFISH data are used for the latter two decades, the increasing trend in the number of exporting countries is stronger (*τ* = 0.51*,* Z_c_ = 2.91, *p* < 0.05).

From the very beginning of the time-series, seafood products have been transported from thousands of kilometres away (Fig. 3b). In the first decade of the twentieth century, imported seafood products travelled on average 2400 kms. In 2020, this had increased to 3500 kms (based on the archive data) or 3900 kms (based on SEAFISH data). The weighted average distance that seafood has been exported from ranged from 1600 kms (minimum, in 1951) to 5300 kms (maximum, in 1962). A weak significant upwards trend in the weighted average distance time-series was identified (modified MK, *τ* = 0.25*,* Z_c_ = 2.24, *p* < 0.05 when using the archive data, or *τ* = 0.27*,* Z_c_ = 2.16, *p* < 0.05 when using SEAFISH data), representing a percentage increase in reach of between 18.2 and 32.3% (Fig. 3b). This suggests that while the UK’s seafood supply network has not greatly expanded (spatially) over the time-series, it has been far-reaching for over a century. The peak in weighted average distance from 1959 to 1969 reflects the peak in fish and marine mammal imports (reaching ~ 732,000 tonnes in 1969). Large quantities of these products were exported from distant nations such as South Africa and to a lesser extent from Peru, Chile, Namibia, Japan and Canada, as well as from Norway, Iceland and Denmark.

### Seafood products

**Fig. 4:**
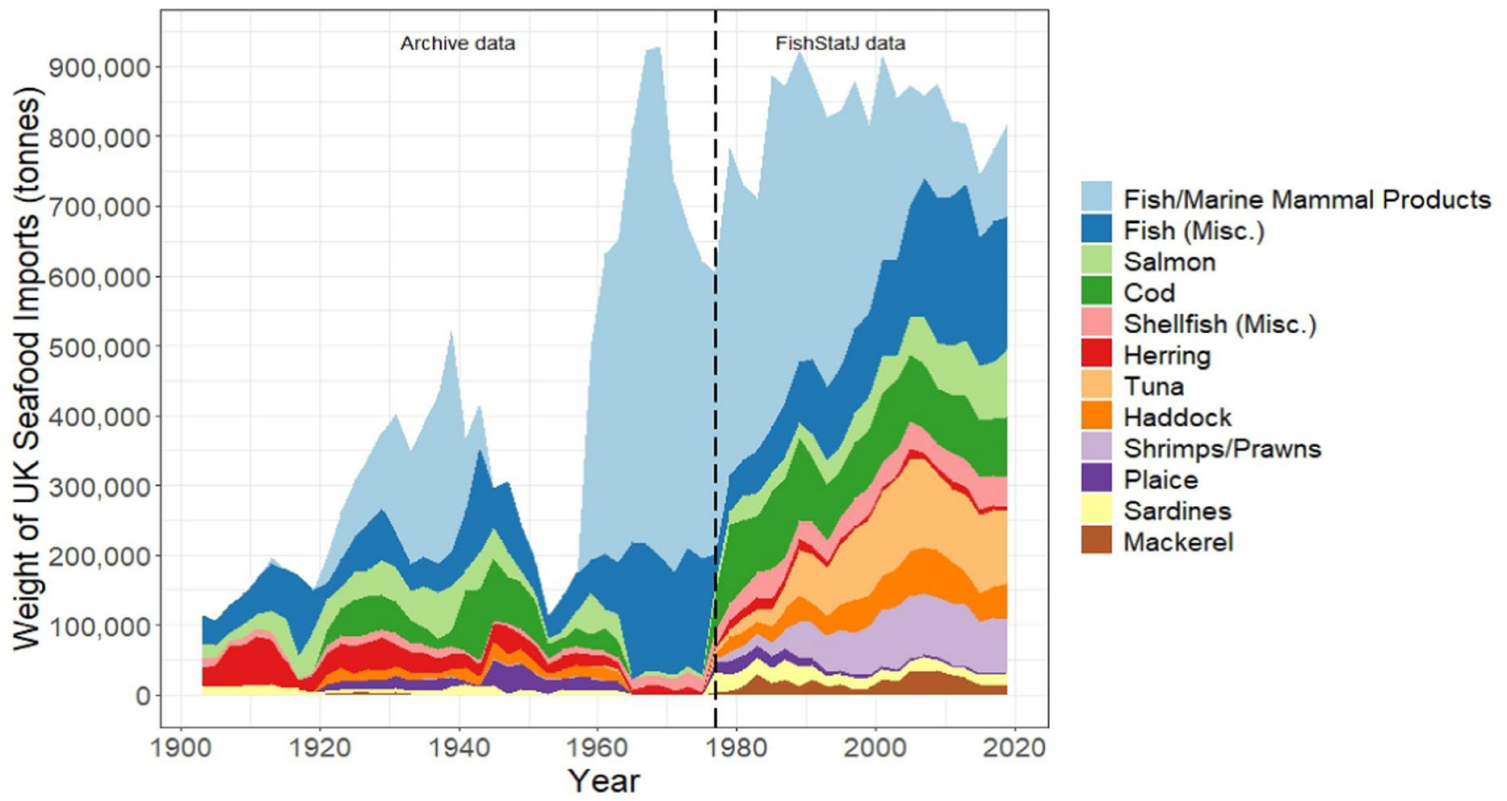
Composition of UK seafood imports by product type (tonnes represent processed weight). Sources: (MMO 2014) from 1903 to 1975, (FAO 2020) from 1977 to 2019

In the first half of the twentieth century, 96% of UK seafood imports consisted of fish/marine mammal products (25%, ~ 3.4 mt), salmon (14%, ~ 1.8 mt), herring (13%, ~ 1.7 mt), cod (12%, ~ 1.5 mt), plaice (4%, ~ 522,000 tonnes), haddock (3%, ~ 432,000 tonnes), and sardines (3%, ~ 398,000 tonnes), as well as large quantities of miscellaneous fish (22%, ~ 3.0 mt) (Fig. 4). The crash in seafood imports from 1945 to 1957 signifies a data gap in the statistical reports, as imports of fish/marine mammal products (fish meals/oils and whale meat/oils) were not recorded (Fig. 4). In the archive data, reported species specificity of imports was reduced from 1965 to 2005, with most seafood products recorded as ‘miscellaneous’ fish and shellfish, except for salmon, herring, and fish/marine mammal products (Fig. 4). From 1965 to 1975, imports predominantly consisted of fish/marine mammal products (74%, 6.1mt tonnes) and miscellaneous fish/shellfish (23%, ~ 1.9 mt tonnes) (Fig. 4). The inclusion of the detailed FishStatJ data (FAO 2020) shows that from 1977 to 2020, key imports again included fish/marine mammal products (35%, ~ 12.6 mt), miscellaneous fish (16%, 5.7 mt,) cod (11%, ~ 3.9 mt), salmon (6%, ~ 2.1 mt) and haddock (5%, ~ 1.7 mt) and imports grew of new popular species such as tuna (10%, ~ 3.6 mt) and shrimps/prawns (7%, ~ 2.6 mt) (Fig. 4).

### The ‘Big 6’

Consumer preference for whitefish, e.g. cod and haddock, has been long-standing and since 1921, tens of thousands of tonnes of these fish were imported annually (Fig. 4). The archival data showed that imports of cod and haddock into the UK came predominantly from wild stocks fished by European nations, namely Iceland, Norway, Demark and the Faroes. However, China became the second largest exporter of cod (after Iceland) from 2007 to 2020, exporting on average over 20,000 tonnes of cod a year (making up on average 18.5% of all cod imports from 2007 to 2020). China was also the third largest exporter of haddock to the UK from 2007 (after Iceland and Norway), exporting on average over 7000 tonnes annually (making up 13.2% of haddock imports from 2007 to 2020).

UK salmon imports were the second largest seafood imports in terms of quantity over the time-series, reaching a peak of nearly 99,000 tonnes in 2019 (Fig. 4). The USA and Canada were the largest suppliers to the UK, followed by Japan, the Faroe Isles and Sweden. Tuna imports grew from ~ 3000 to 4000 tonnes/annum in the early 1960s, to ~ 10,000–120,000 tonnes/annum from 1977 (Fig. 4). The UK has depended largely on island nations to meet demand for tuna products, principally Mauritius and the Seychelles, and to a lesser extent Ghana, Thailand and the Philippines. Shrimps and prawns also became amongst the most heavily traded seafood commodities, with the UK importing from ~ 10,000 to 90,000 tonnes annually since 1977 (Fig. 4). Shrimp/prawn imports made up on average 57.4% of total shellfish imports into the UK from 1997 to 2020. The UK imported warmwater species primarily from India, Thailand and Vietnam and sourced cold-water species primarily from Iceland and Denmark. Fish and marine mammal products were the largest imports by volume into the UK, with the top five exporters being Norway, Iceland, Denmark, South Africa and Peru. Imports of fish/marine mammal products reached a peak of over 320,000 tonnes in 1939 and peaked again at over 730,000 tonnes in 1969, exceeding imports of seafood for direct human consumption from the late-1950s to the mid-1970s (Fig. 4).

## Discussion

### The UK’s seafood footprint

Research on fisheries development commonly focuses on localised fishery landings and effort data, with only a subset of researchers considering longer-term (multi-decadal or centennial) patterns (e.g. from the ‘Sea Around Us’ research initiative and the ICES ‘Working Group on the History of Fish and Fisheries’). Our analysis of 120-years of UK data focuses on a lesser considered aspect, the fish products available from imports, and the corresponding global footprint of the UK. Our data shows that, for as long as national records have existed, British consumption patterns have been supplemented and shaped by fish sourced from countries thousands of kilometres away. Over time, the volume of seafood products imported expanded, as did the distance travelled by imports. In contrast to prevailing fisheries policy which focuses on national and European-scales (Huggins et al. 2019; Mardle et al. 2002; Symes 1992), this wider perspective shows that seafood demand has expanded far beyond these jurisdictional borders for more than a century.

The UK’s seafood imports grew over six-fold in volume from 1900 to 2020. During the World Wars, imports supported greatly reduced domestic supplies (Harrison et al. 2023; Kerby et al. 2012); pre-war fish import duties were suspended so that foreign countries in a position to send fish would be free to supply as much as they could (Ministry of Agriculture and Fisheries 1946). Increased import demand over WW2 can be observed as a sharp but short-lived upwards trend in seafood imports, overtaking domestic landings (Fig. 1). Domestic landings rebounded after World War II as local fishing grounds had the chance to recover, hence lessening the demand for foreign seafood products. There was a pronounced increase in UK landings from the late 1940s to the early 1970s and a corresponding dip in imports from the late 1940s to the late 1950s (Fig. 1), though this may also be attributed to the change in reporting style of import data, whereby imports of fish and marine mammal products were not included from 1945 to 1959. Imports of fish/marine mammal feedstock products after this period exhibited a notable and consistent upward trend (Fig. 4), coincident with the rise in commercial aquaculture production in the UK (Green 2012; Liu and Sumaila 2008; Thurstan and Roberts 2014). The UK’s growing dependence on seafood imports in the latter twentieth century (Fig. 1) is primarily a result of rising demand for seafood and subsequent depletion of commercial food fish stocks in the North Sea and North Atlantic over the twentieth century (Alheit et al. 2005; Myers et al. 1996; Thurstan et al. 2010), declines in domestic fishery landings in the late twentieth century, and the imposition of progressively restrictive fishing regulations from the 1980s (Harrison et al. 2023). Restricted fishing opportunities, especially for species historically preferred by UK consumers, ultimately led to the UK’s growing dependence on seafood imports to meet ongoing demand (Harrison et al. 2023), evidenced by imports exceeding domestic seafood production from 1985 (Fig. 1).

Seafood imports not only increased in volume but expanded spatially over the time-series by between 18.2 and 32.3% in terms of kilometres travelled (Fig. 3b). From 1900 to 1950, over 90% of the UK’s seafood imports were produced by European countries, Russia and North America, with imports primarily consisting of traditionally favoured cold-water species such as salmon, cod, herring and plaice, as well as fish/marine mammal products used in agri/aquaculture industries. Other more distant countries, primarily under British occupation or governance at the time, also contributed to meeting demand in the early twentieth century. Substantial changes to the origin of seafood supplies were most notable from the 1950s when dominant exporting nations expanded to cover all continents (Fig. 2), confirming the lengthening of supply chains and expansion of global seafood trade networks from the mid-twentieth century (International Transport Forum 2015; Taylor et al. 2007). The expansion of seafood trade networks allowed the UK to more effectively outsource fisheries production and capitalise on cheap and plentiful commodity fish exports from the Global South (Belton et al. 2020). This was facilitated in-part by the UK’s accession to the European Common Market in 1973 and the subsequent creation of the Lomé Convention in 1975 which provided a framework for preferential trade with developing countries in Africa, the Caribbean and the Pacific, specifically with former European colonies (CVCE 2017; Swartz et al. 2010). UK-EU trade relations have historically been pivotal in shaping the seafood supply chain within Europe and beyond (Symes and Phillipson 2019). However, after the UK departed from the European Union in 2020, the nation faces a barrage of increased trade barriers, tariff restrictions and altered trade relations with EU countries (Halevy and Trewern 2023; Symes and Phillipson 2019). A report by the Confederation of British Industry found that direct imports into the UK from developing countries, in particular from Vietnam and Ecuador, increased by 15% following Brexit in 2020 (CBI 2021).

Food production is responsible for a quarter of anthropogenic greenhouse gas emissions, with transportation and final delivery from producer to retailer also contributing a large proportion (Aragão et al. 2022; Kristofersson et al. 2021; Parker et al. 2018; Weber and Matthews 2008). Whilst fishing and aquaculture produce protein with far lower emissions per unit of output compared to land-based animal proteins (Hilborn et al. 2018; Turrell 2019; Weber and Matthews 2008), emissions from the fishing industry grew by 28% from 1990 to 2011 due to energy intensive fishery technological advances and seafood processing/storage, increased use of fuel and lower catch per effort, with fishing now contributing approximately 4% of global food production emissions (Feng et al. 2024; Kristofersson et al. 2021). More cumulative product being exported over greater distances, either to reach their final destination or for intermediary supply chain steps (i.e., for processing), will have also inevitably led to a rise in carbon emissions from freight transportation owing to increased fuel consumption and energy expenditure for refrigeration (Madin & Macreadie 2015; Parker et al. 2018). Maritime transportation, responsible for transporting 80% of global trade, has long been a significant contributor to global carbon emissions (~ 2.5–3%), and emissions from shipping have fluctuated on an upwards trend over the past two decades, mirroring the growing global freight trade (Feng et al. 2024). Increases in transportation emissions have also been exacerbated by the use of air freight (Aragão et al. 2022), which contributes ~ 170–200 times more emissions than transport via ship (Eurofish 2014). In the 1970’s air freighters such as the Boeing 747 came into use (Bañez et al. 2019) and became responsible for transporting ~ 10% of seafood cargo internationally (CEVA Logistics 2023). Whilst ~ 60–85% of air cargo is transported on passenger aircraft (and the rest via cargo planes), the additional weight on board increases kerosene consumption and fuel combustion (Eurofish 2014; FTA 2015). It can be inferred that the UK’s dependence on imports, and the globalisation of seafood trade networks more widely, has led to the continued growth of seafood carbon intensity (Feng et al. 2024).

UK Sea Fisheries Statistics and SEAFISH data were not sufficient to accurately determine the geographical source of fish products since they reported the country of dispatch rather than the raw material source, nor did they report on the intermediary supply chain steps, e.g., for processing (WWF 2022). It is therefore difficult to ascertain the full extent of the UK’s global footprint which has likely been much greater than presented in this study. For example, countries such as China, Russia, Japan and Spain dominated distant-water fishing in the high seas, as well as in other countries EEZs (often illegally), over the course of the twentieth century, thus limiting our ability to determine the source of the seafood products these nations then export (Urbina 2023). Imports of marine mammal products from European countries (primarily Norway), the Soviet Union, South Africa, the Americas and Japan likely came from Antarctic waters as these nations were whaling here across the twentieth century (Schneider and Pearce 2004). Furthermore, in modern supply chains seafood products are transported and processed between a number of countries before reaching destination markets (Asche et al. 2022; Fox et al. 2018). The ‘re-exporting’ phenomenon, which confuses the country of origin for the country of processing, packaging and re-exporting, prevents assignment of imports to their provenance (Asche et al. 2022). China, a country that the UK has become increasingly reliant upon to meet seafood demand (Fig. 2), became the world’s largest seafood producer, processor and re-exporter from the late twentieth century, sourcing fish from countries around the world and re-exporting ~ 75% of them as products of China (Asche et al. 2022; Wolf et al. 2020). This phenomenon presents salient sustainability concerns as at each stage of handling, industries have the opportunity to change the identity, purity and authenticity of the seafood product for financial gain (Asche et al. 2022; Fox et al. 2018; Kroetz et al. 2020). Modern DNA forensics testing is revealing the prevalence of deliberate mislabelling and attribution in supply chains (Luque and Donlan 2019; Naaum et al. 2016).

### Socio-ecological repercussions

Increased imports of seafood products to supplement shortfalls in domestic landings has been shown to facilitate economic growth, create jobs and contribute to poverty and malnutrition alleviation in exporting countries (Asche et al. 2015; FAO 2022; Watson et al. 2017). However, profound environmental and social costs also arise from such exports, many of which are hidden from the consumer and difficult to quantify (Belton et al. 2020). Some of the most important are discussed below.

Many fishery product cycles in the Global South are at a transitional stage of development and are characterised by spatial expansion, intensification and nascent regulation (Alder and Watson 2007; Belton et al. 2020). They are also driven largely by commoditisation, that is, the drive to produce more fish for expanding local markets and for export (Belton et al. 2020). Economic interdependencies fostered through international trade, coupled with limited governance capacity, has meant a replay of problems experienced earlier by developed country fisheries, viz., fishery overcapacity, increased prevalence of illegal, unreported and unregulated (IUU) fishing, the use of unselective/destructive fishing methods, environmental degradation, and wild fish stock declines (Alder and Watson 2007; Asche et al. 2015; Belton et al. 2020; Gephart and Pace 2015; Graziano et al. 2018; Sampson et al. 2015; Ye and Gutierrez 2017). For example, demand for shrimp induced a supply response in the Global South (e.g., in India, Thailand and Vietnam) where fishing for export intensified, achieved by the geographical expansion of capture fisheries effort and the promotion of intensive farming operations (Belton et al. 2020; FAO 2022; Oudwater et al. 2002). While expansion of capture fisheries has amplified the use of bottom-towed gear which has a considerable impact on seabed habitats, shrimp farming has also expedited large-scale destruction of critical coastal habitats such as mangroves, leading to the demise of a multiplicity of social and ecological systems (See: Alder and Watson 2007; Alongi 2002; Azad et al. 2009; Thurstan and Roberts 2014).

Another highly commoditised and globally traded seafood product is fishmeal. Fishmeals were used for agricultural purposes and high-protein livestock and poultry feed, and later to feed the aquaculture industry (FAO 2022). The intensification of aquaculture operations from the 1960s fuelled the rise of highly destructive reduction fishing industries, using fine mesh bottom trawl nets which indiscriminately catch low value ‘trash fish’- often juveniles, or vast pelagic nets catching forage fish species such as Peruvian anchoveta, before rendering down into fishmeal and oil (Asche and Tveterås, 2004; Belton and Thilsted 2014; Myo et al. 2018). This feed is then fed to high value aquaculture products such as prawns and salmon, thereby converting low-value catch into a profitable product, at an enormous and unsustainable environmental cost if the reduction fish stocks are ungoverned (Yonmo 2021). However, the adoption of sustainable fisheries management systems over the past few decades (e.g., improved regulation of trawl fisheries in SE Asia and strict catch limits and seasonal fishery closures in Chile, Peru, the USA and Nordic countries) (Asche and Tveterås, 2004; Shepherd and Jackson 2013), coupled with the falling of aquaculture feed conversion ratios due to better understanding of nutrition requirements and improved feed management (Glencross et al. 2023) and the rise of alternative and more economical fishmeal ingredients (e.g., plant-based feed alternatives), have alleviated pressure on wild reduction fisheries (Naylor et al. 2009).

Social repercussions of fisheries production for export are multifaceted, for instance, the diversion of essential protein away from local communities (Alder and Watson 2007; Rood and Schechter 2007). Fisheries in less developed economies are increasingly focusing on supplying lucrative species, such as salmon, shrimps and tuna for export and focusing less on catching lower-value species eaten by locals (FAO 2022; Rood and Schechter 2007). Likewise, the reduction of increasing amounts of catches of whole edible fish into fish meals/oils for export, which were previously used for direct human consumption, is impacting food security namely in West African countries (FAO 2022). The global demand for high value species such as warm water prawns, tuna and squid has also led to the prevalence of human rights abuses such as forced labour in fisheries especially across Asia (WWF 2022). Labour trafficking has been documented on American, South Korean and Thai boats, with China being the worst perpetrator of violence, poor working conditions and debt bondage (Urbina 2023).

Concerted efforts from government and retailers will be critical to improve domestic seafood production and reduce exporting developed nations’ environmental footprints to other countries, as the UK has done for over a century. The UK should encourage better standards for seafood trade transparency (Asche et al. 2022) and require reliable sustainable certification of imported products (Sampson et al. 2015). This is especially relevant as the UK has become increasingly reliant on China to meet consumer demand, a country that has come under scrutiny for widespread illegal fishing, sourcing fish from IUU fisheries, mislabelling and re-exporting, and human rights abuses. Large-scale cultural norms must also be addressed, as habitual consumer behaviour will perpetuate the spatial expansion and intensification of fishing pressure for popular products if not subject to strict precautionary management. The UK should consider how local demand for seafood has resonated at a global level, especially the wider environmental costs.

## Conclusions

Drawing on historical trade data from the 1900s has provided an opportunity to explore the evolution of the UK’s global seafood import footprint. The UK experienced major changes to domestic fisheries sectors, with increased demand and fishing pressure leading to local collapses of commercial fish stocks in the twentieth century, followed by major policy changes. Meanwhile, habitual consumer behaviours had developed and demand for popular food fishes, such as the ‘big 5’, in-turn led to reduced self-sufficiency and a growing dependence on seafood imports from a growing number of countries. The social and ecological costs of this reliance are multifaceted and severe. International trade has shifted fishing pressure elsewhere and in many cases led to export of overfishing and depletion of distant stocks (Berkes et al. 2006; Worm and Branch 2012). Carbon emissions related to increased fishing activity and increases in freight transportation distances must also be considered as an environmental cost, especially as the UK becomes increasingly dependent on seafood from further afield post-Brexit. Attempting to quantify the ecological and social impacts of import demand on exporting countries, and calculating the carbon footprint of imported seafood products are both noteworthy avenues for future research.

## Supplementary Information

Below is the link to the electronic supplementary material.

Supplementary file1 (PDF 222 KB)

## Data Availability

The data that support the findings of this study are available in Figshare using the identifier: https://doi.org/10.6084/m9.figshare.25690347.
