# Supplementary material for: The UK’s expanding global reach for seafood over 120-years
Source: Rev Fish Biol Fish. 2025 Apr 18;35(2):943–61. doi: 10.1007/s11160-025-09942-x (PMC12065761; doi:10.1007/s11160-025-09942-x)
Supplement: Supplementary file 1 — Supplementary file1 (PDF 222 KB) [file 11160_2025_9942_MOESM1_ESM.pdf]

Online Resource 1

The UK's expanding global reach for seafood over 120-years
Reviews in Fish Biology and Fisheries
Zoe F.J. Heard[1], Callum M. Roberts[1], Ruth H. Thurstan[1]
[1] Centre for Ecology and Conservation, University of Exeter, Cornwall, UK, TR10 9FE
Corresponding author: zh263@exeter.ac.uk

Comparison of top 10 product-specific imports (and 'misc' fish and shellfish imports) into the UK from archive (UK Sea Fisheries Statistics) data, FishstatJ data and SEAFISH data.

- Product specificity of UK imports was reduced in the archival data from 1965-2005 (i.e., most species were recorded as miscellaneous fish or shellfish products), except for herring, salmon and fish/marine mammal products. Hence, comparison between the FishStatJ data and archive data was only possible from 2007 for most products. From 2011, the archive and FishStatJ data could be compared to the SEAFISH data.
- The reason for reduced species-specificity of seafood imports by country of export in the archive data is unclear and may be attributed to changes in reporting style of the annual reports.
- Plaice and herring imports were not recorded in the archive data from 2007 due to being imported in relatively low quantities (>10,000 tonnes).
- Fish/marine mammal product imports were not recorded in the SEAFISH data, likely as these were not considered as edible 'seafood'.
- The reasons for small discrepancies between the data sources, e.g., for cod, salmon, mackerel, sardines and tuna are unclear.

Average difference between archive data and FishStatJ data (including misc. fish/misc. shellfish import data from 2007) **= 12.4%**
Average difference between SEAFISH data and FishStatJ data (excluding fish/marine mammal product imports) **= 13.7%**

Comparison plots of UK product-specific imports (in tonnes), across the three data sources, are shown below. The orange line represents the archive data, the blue line represents FishStatJ data, and the green line represents the SEAFISH data.

Key:

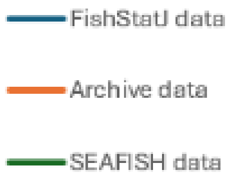

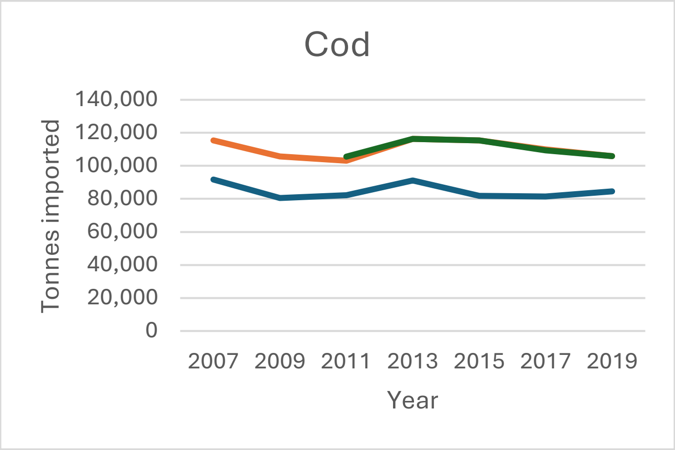

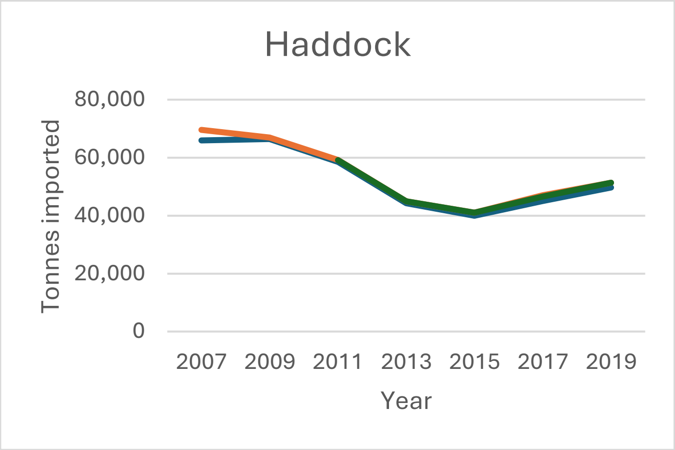

Average difference: FishStatJ & Archive data= 30%
FishStatJ & SEAFISH= 31%

Av. Difference: FishStatJ & Archive data=3%
FishStatJ & SEAFISH = 2%

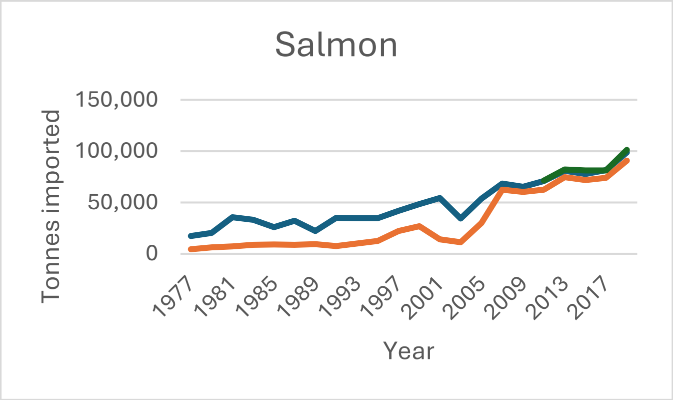

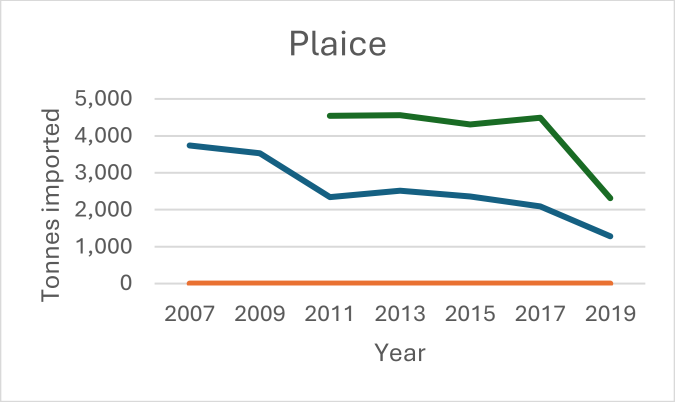

Av. Difference: FishStatJ & Archive data= 47%
FishStatJ & SEAFISH= 2%

Av. Difference: FishStatJ & Archive data=100%
FishStatJ & SEAFISH= 91%

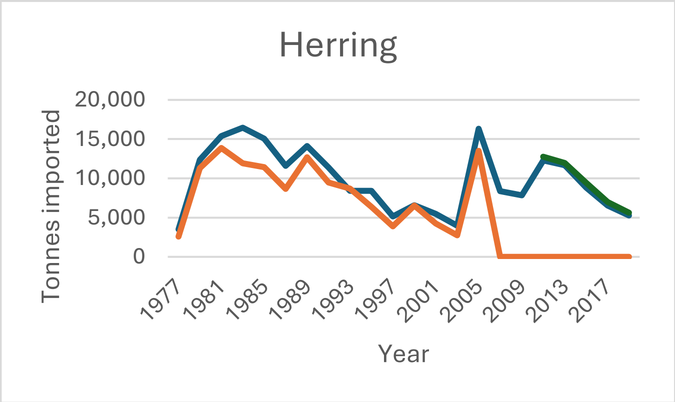

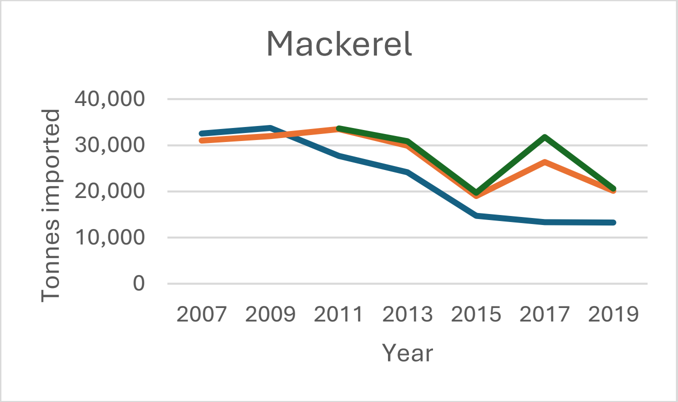

Av. Difference: FishStatJ & Archive data= 44%
FishStatJ & SEAFISH= 6%

Av. Difference: FishStatJ & Archive data= 31%
FishStatJ & SEAFISH = 55%

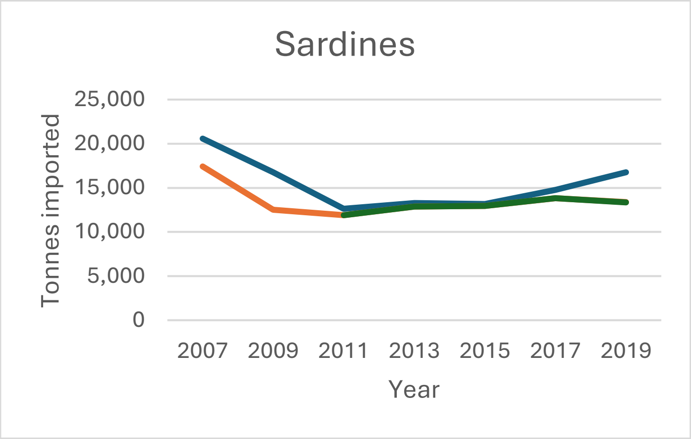

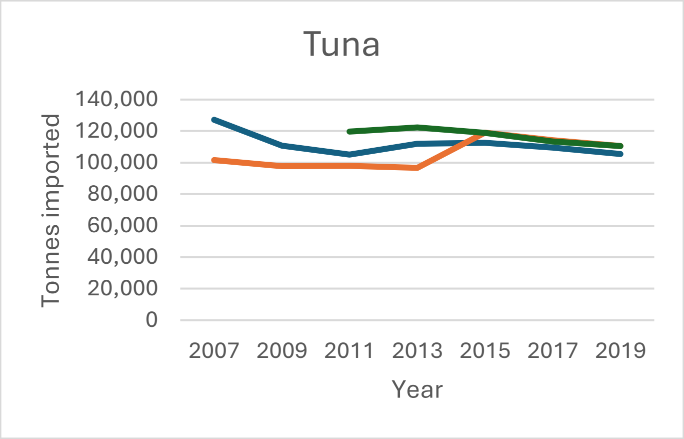

Average difference: FishStatJ & Archive data= 11%
FishStatJ & SEAFISH= 7%

Av. Difference: FishStatJ & Archive data=5%
FishStatJ & SEAFISH = 7%

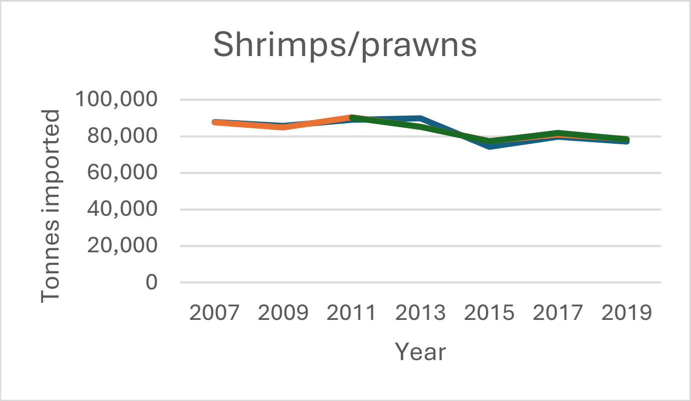

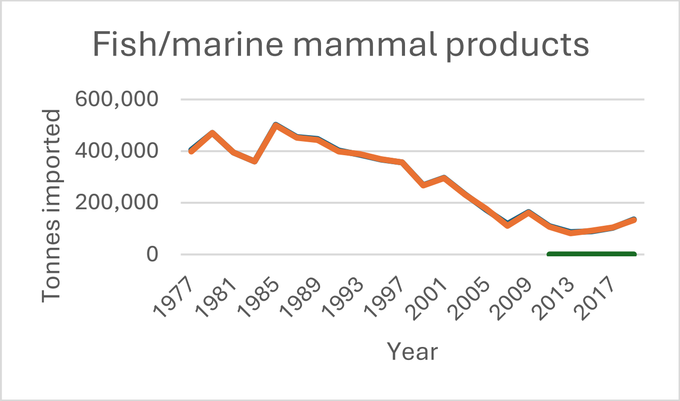

Av. Difference: FishStatJ & Archive data= 0%
FishStatJ & SEAFISH= 1%

Av. Difference: FishStatJ & Archive data=1%
FishStatJ & SEAFISH= n/a

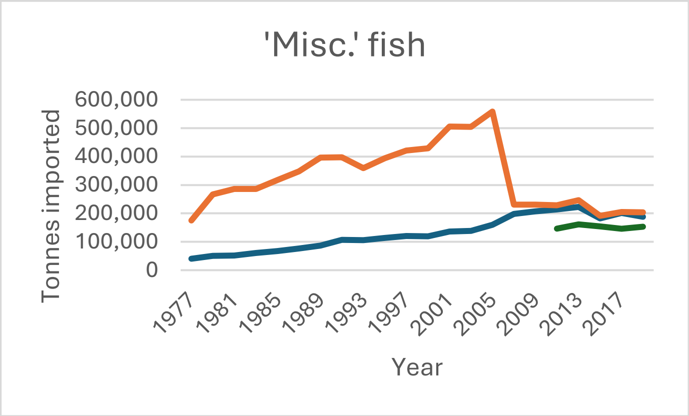

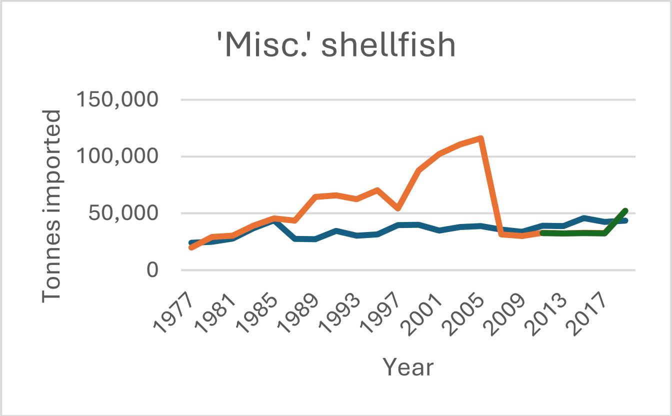

Av. Difference: FishStatJ & Archive data (from 2007)= 9%
FishStatJ & SEAFISH= 24%

Av. Difference: FishStatJ & Archive data (from 2007)= 12%
FishStatJ & SEAFISH= 13%
